# Antioxidant Dimethyl Fumarate Temporarily but Not Chronically Improves Intracortical Microelectrode Performance

**DOI:** 10.3390/mi14101902

**Published:** 2023-10-04

**Authors:** George F. Hoeferlin, Tejas Bajwa, Hannah Olivares, Jichu Zhang, Lindsey N. Druschel, Brandon S. Sturgill, Michael Sobota, Pierce Boucher, Jonathan Duncan, Ana G. Hernandez-Reynoso, Stuart F. Cogan, Joseph J. Pancrazio, Jeffrey R. Capadona

**Affiliations:** 1 Department of Biomedical Engineering, Case Western Reserve University, 10900 Euclid Ave, Cleveland, OH 44106, USAhjo10@case.edu (H.O.); jld155@case.edu (J.D.); 2 Advanced Platform Technology Center, Louis Stokes Cleveland Veterans Affairs Medical Center, 10701 East Blvd, Cleveland, OH 44106, USA; 3 Department of Bioengineering, The University of Texas at Dallas, 800 W Campbell Rd, Richardson, TX 75080, USAjoseph.pancrazio@utdallas.edu (J.J.P.)

**Keywords:** intracortical microelectrode arrays, neuroinflammation, dimethyl fumarate, antioxidant, neural engineering, brain-machine interface, neural interface

## Abstract

Intracortical microelectrode arrays (MEAs) can be used in a range of applications, from basic neuroscience research to providing an intimate interface with the brain as part of a brain-computer interface (BCI) system aimed at restoring function for people living with neurological disorders or injuries. Unfortunately, MEAs tend to fail prematurely, leading to a loss in functionality for many applications. An important contributing factor in MEA failure is oxidative stress resulting from chronically inflammatory-activated microglia and macrophages releasing reactive oxygen species (ROS) around the implant site. Antioxidants offer a means for mitigating oxidative stress and improving tissue health and MEA performance. Here, we investigate using the clinically available antioxidant dimethyl fumarate (DMF) to reduce the neuroinflammatory response and improve MEA performance in a rat MEA model. Daily treatment of DMF for 16 weeks resulted in a significant improvement in the recording capabilities of MEA devices during the sub-chronic (Weeks 5–11) phase (42% active electrode yield vs. 35% for control). However, these sub-chronic improvements were lost in the chronic implantation phase, as a more exacerbated neuroinflammatory response occurs in DMF-treated animals by 16 weeks post-implantation. Yet, neuroinflammation was indiscriminate between treatment and control groups during the sub-chronic phase. Although worse for chronic use, a temporary improvement (<12 weeks) in MEA performance is meaningful. Providing short-term improvement to MEA devices using DMF can allow for improved use for limited-duration studies. Further efforts should be taken to explore the mechanism behind a worsened neuroinflammatory response at the 16-week time point for DMF-treated animals and assess its usefulness for specific applications.

## 1. Introduction

Intracortical microelectrode arrays (MEAs) are an integral part of brain-computer interface (BCI) systems that allow for motor restoration in people with spinal cord injury, can offer an approach to reduce the burden of treatments for various neurological disorders, and are used to further understand the complexity of brain functions [1,2,3,4,5]. Unfortunately, MEAs tend to fail over weeks to months following implantation, limiting their utility in many applications [6,7,8,9,10]. Consequently, there is a need for new designs and treatments to prolong the function of MEAs. Specifically, new developments need to be made to address the biological response from the body triggered by an implanted MEA.

Upon implantation, MEAs can rupture blood vessels, causing damage to the blood-brain barrier (BBB) and damaging nearby neurons [11,12,13,14]. Within minutes [8,15,16], microglia in the brain and infiltrating macrophages surround the MEA device and become activated via innate immunity pathways [17,18,19,20,21,22]. For the duration of the implant, microglia and macrophages are activated, presumably producing pro-inflammatory cytokines and chemokines and releasing reactive oxygen species (ROS) around the implant site that further damage the surrounding tissue [23,24,25,26,27,28]. Over weeks, astrocytes activate and migrate around the MEAs to form a glial scar to isolate the device from the rest of the brain [29,30]. As a result, the biological response can contribute to MEA failure, consisting of a loss of recording function.

One factor contributing to the perpetual biological response is the constant presence of ROS and subsequent production from immune cells. As a byproduct of immune cell metabolism, ROS are released into the surrounding tissue when generation in microglia and macrophages exceeds the ability for natural removal [31,32,33]. ROS are highly reactive molecules that may trigger oxidative cellular damage and death to neurons and tissue surrounding the implant [34,35]. Limiting the amount of ROS after implantation offers a potential solution to reducing cellular damage and improving neural recordings.

Our team has focused on improving MEA function and tissue health using antioxidant therapeutics to reduce ROS accumulation around the implant site. Systemic and local delivery of the antioxidants resveratrol and curcumin have shown promise in reducing neuroinflammation and improving MEA performance [24,26,36]. Additionally, we have developed an antioxidant coating of the superoxide dismutase mimetic Mn(III)tetrakis(4-benzoic acid)porphyrin (MnTBAP) that shows reductions in oxidative stress and improved MEA performance [37,38].

Here, we aimed to further the understanding of novel antioxidant therapeutics for improving MEA performance using dimethyl fumarate (DMF). DMF (Tecfidera) is FDA-approved for treating multiple sclerosis due to its neuroprotective and antioxidant effects [39,40]. It has been shown to mitigate damage in rodent models of neurological disorders, including traumatic brain injury [41,42,43,44,45,46,47]. As MEA implantation may be considered a form of focal traumatic brain injury, we hypothesized that DMF could alleviate and improve the biological response after device implantation. DMF increases Nrf2 pathway activity, a transcription factor known as a master regulator of antioxidant abilities [48,49,50]. Furthermore, DMF and its biologically active metabolite, monomethyl fumarate (MMF), can cross the blood-brain barrier (BBB) at pharmacologically relevant levels, as indicated by direct effects on tissue and cells for inducing antioxidant effects [51,52,53]. Here, we investigate the effect of DMF on the neuroinflammatory and oxidative stress response at both a sub-chronic (7 weeks) and a chronic time point (16 weeks post-implantation) and MEA recording performance across acute (1–5 weeks), sub-chronic (6–11 weeks), and chronic (12–16 weeks) neuroinflammatory phases.

## 2. Materials and Methods

### 2.1. Intracortical Microelectrode Array Preparation

Before implantation, the 16-channel (electrode), single-shank intracortical microelectrode array (A1x16-3 mm-100-177-Z16, iridium electrode sites, NeuroNexus Technologies, Ann Arbor, MI, USA) quality was verified via electrochemical impedance spectroscopy (EIS) testing. In a Faraday cage, each MEA to be implanted underwent EIS testing with a Gamry Interface 1010E Potentiostat (Gamry Instruments, Warminster, PA, USA) consisting of each electrode site as the working electrode, a platinum wire as a counter electrode, and an Ag|AgCl reference electrode for measurements. EIS was performed in 1× phosphate-buffered saline (PBS) (pH = 7.4) over 1 to 10^6^ Hz (12 points per decade) with an AC voltage of 50 mV rms. Device verification was determined by measuring the impedance value at 1 kHz. If a device was above 1 MΩ or below 100 kΩ, it was not used for implantation. Following EIS verification, MEAs were cleaned by dipping in 70% ethanol and DI water to remove any residual 1× PBS and optically imaged using a Keyence Optical Microscope (Keyence Corporation, Osaka, Japan) at a magnification of 150× for visual inspection. MEAs were then sterilized under cold-gas ethylene oxide sterilization for surgical use. 

### 2.2. Animals and Surgical Implantation

**Figure 1:**
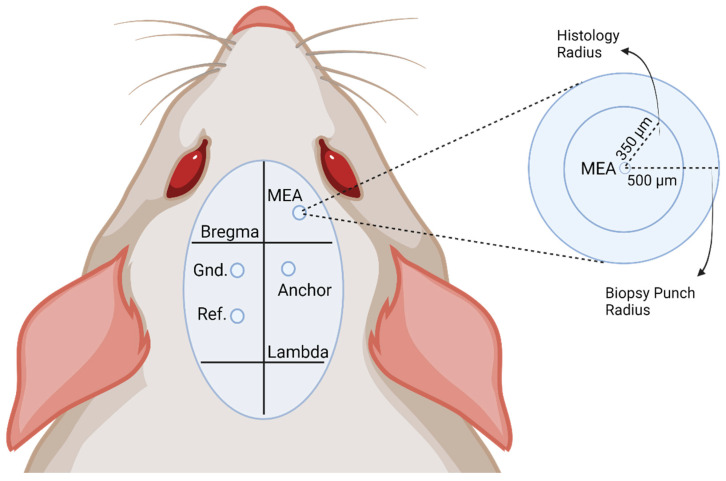
Schematic detailing implantation details. The labels on the skull indicate the location at which each craniotomy is performed on the skull for the MEA device and the ground (Gnd.), reference (Ref.), and anchor screws. A blow-up view of the implant site is provided to clarify the distance from the implant to the respective analysis method. Histological analysis is performed within a 350 µm radius, while gene analysis is conducted within a 500 µm radius of the implant.

The Louis Stokes Cleveland Department of Veteran Affairs Medical Center Institutional Animal Care and Use Committee approved all animal work outlined. Surgical procedures follow those of previously established protocols [38]. Briefly, 35 Sprague Dawley rats (Charles River Labs, Wilmington, MA, USA) aged 8–10 weeks old were anesthetized in an isoflurane chamber (3.5% isoflurane in O_2_ at 1.5 L/min). Of the 35 rats, 22 were implanted with functional devices for the 16-week recording study (11 for control and 11 for DMF treatment) and 13 non-functional “dummy” probes for the 7-week genomic assessment of the neuroinflammatory response (7 for control and 6 for DMF treatment). For all surgeries, anesthesia level was monitored by paw-pinch reflex and assessing drowsiness by tracking the animal’s movement. Eye ointment was applied to keep the eyes from drying. The incision site on the animal’s head was shaved clean and nails clipped to prevent ripping out of sutures during recovery. The rat was then mounted onto a stereotaxic frame (David Kopf Instruments, Tujunga, CA, USA), and anesthesia was maintained via isoflurane delivery at 0.5–2.0% isoflurane in O_2_ at 1.5 L/min via inhalation through a nose cone. The animal’s vital signs were monitored and maintained via a blood-oxygenation and heart rate measurement system with a warming pad (PhysioSuite^®^ for Mice and Rats, Kent Scientific Corporation, Torrington, CT, USA). A single injection of Marcaine (0.15 mL of 2.5 mg / mL stock concentration) was administered subcutaneously to the incision site. A subcutaneous injection of the analgesic Buprenorphine (0.05 mg/kg body weight) was administered by the scruff of the neck to help with pain and recovery. The surgical area was scrubbed with alternating chlorohexidine gluconate and 70% isopropanol swabs. A one-inch incision was made along the scalp midline, and the underlying connective tissue or muscle was removed to expose the skull. Sterile alligator clips were used to pull back any excess skin. A swab of hydrogen peroxide was used on the skull to dry out the surface and make the cranial sutures visible, including the bregma and lambda points. A thin coat of Vetbond tissue adhesive (Catalog #70200742529, 3 M, Saint Paul, MN, USA) was applied to the clean skull to prime the surface for later cement application. For the functional-implanted rats, craniotomies for three stainless steel bone screws (Stoelting Co., Wood Dale, IL, USA) were hand-drilled into the skull using a 1.35 mm drill bit (see Figure 1 for schematic) [54,55]. A ground screw (−1.5 mm lateral to midline and −1.5 mm posterior to bregma), a reference screw (−1.5 mm lateral, −4.5 mm posterior to bregma), and an anchoring screw (1.5 mm lateral, −3 mm posterior to bregma) were inserted into the skull to act as ground and a reference for electrophysiological recordings (only in the animals that received recording electrodes). A final craniotomy for the implant site was drilled using a 1.75 mm drill bit located at 2 mm lateral and 2 mm anterior to the bregma, targeting the primary motor cortex (M1). For the non-functional “dummy” probes, a single craniotomy was drilled using a 1.35 mm drill bit at 2 mm lateral and 2 mm anterior to the bregma. In both cohorts, the implant was ready for insertion once the dura was removed and the brain was clear of any dura or debris. The intracortical microelectrode was positioned above the craniotomy to avoid blood vessels. For the functional implant cohort, reference and ground wires for the device were wrapped around their bone screws before implantation. Once positioned, the MEA was implanted into the motor cortex to a depth of 2 mm via a hydraulic inserter (2650 Micropositioner, David Kopf Instruments, Tujunga, CA, USA). For the non-functional “dummy” probe cohort, the NeuralGlider (Actuated Medical, Bellefonte, PA, USA) inserter was used to insert the probe at a depth of 1.3 mm. Non-functional probes were not inserted at a depth of 2 mm due to the length of the probe. For both cohorts, a dural graft (Biodesign Dural Graft, Cook Medical, Bloomington, IN, USA) was placed around the craniotomy to promote healing, followed by a layer of Kwik-Sil silicone adhesive (World Precision Instruments, Sarasota, FL, USA) to seal the craniotomy site. Teets Cold Cure dental cement (A-M Systems, Sequim, WA, USA) was then applied around all the craniotomy sites to build up an anchored dental cement head cap to secure the implant into place. Following surgery, 5–0 monofilament polypropylene sutures were used to close the surgical site for proper healing while leaving enough skin not sutured to keep the electrode connector exposed. A one-time dose of antibiotic Cefazolin (5 mg/kg, subcutaneously) was given immediately after surgery, followed by a continuous dose of antibiotics (Trimethoprim-Sulfamethoxazole oral suspension, 53 mg/kg/24 h) in the animal’s drinking water for up to 7 days. Twice daily, animals received buprenorphine (0.05 mg/kg, subcutaneously) for up to 72 h to treat pain.

### 2.3. Drug Preparation and Delivery

The vehicle for drug delivery was chosen based on past literature [41,42,56] and formulated as 8% Methocel solution (Methocel^®^ A15 LV, Catalog #64605, Millipore Sigma, Burlington, MA, USA) in DI water (8 g Methocel dissolved in 100 mL DI water), hereby referred to as 8% Methocel solution. Methocel acts as a suitable suspending agent for orally delivering DMF. The Methocel vehicle was aliquoted into 20 mL containers for either control or treatment preparation. For treatment, 97% Dimethyl Fumarate (DMF, Catalog #242926, Millipore Sigma, Burlington, MA, USA) was prepared by grinding into a fine powder via mortar and pestle. Powdered DMF was then measured at a concentration of 60 mg DMF per 1 mL of an 8% Methocel solution. Following sufficient suspension using a planetary mixer, the DMF/Methocel solution was then drawn up in 1 mL syringes to be frozen and stored in a −80 °C freezer until the day of treatment. Another batch was prepared consisting of only 8% Methocel with no DMF to act as the vehicle treatment for the control group and frozen until the day of treatment. Each week for 16 weeks, every rat was weighed to determine the necessary DMF delivery to match a 60 mg/kg body weight dosing. During the day of treatment, DMF and control treatment syringes were taken from the freezer and allowed to thaw to room temperature before oral gavage treatment occurred. The control groups were given 8% Methocel at the same volume as if they were given a 60 mg/kg body weight dose to stay consistent. Due to the fast metabolism of DMF, a 1× daily oral gavage delivery of either DMF or vehicle control was required to maintain clinically relevant levels of DMF. Oral gavage provides accurate dosing to ensure the total dose of DMF is obtained by each animal and matches that of clinical trials in which clinically available DMF is provided for oral use in pill form. 

### 2.4. Neurophysiological Recordings

Electrophysiological recordings were acquired from the MEA devices twice weekly for the 16-week study, including a recording taken on the same day as surgery once the animal had awoken (Day 0). Animals were placed under isoflurane anesthesia (3.5% isoflurane in O_2_ at 1.5 L/min) for each recording session until unresponsive [57,58,59]. Animals were then placed in an acrylic box inside a Faraday cage with recording equipment to shield them from outside electromagnetic interference. The MEA connector was clipped into a 16-channel ZIF-Clip Head stage (Tucker-Davis Technologies Inc., Alachua, FL, USA) that was part of a 32-channel motorized commutator system (Catalog #ACO32, Tucker-Davis Technologies Inc.) to allow for the animal to roam freely without getting caught on the cord [18,21]. The commutator was connected to a RA16PA 16-channel Medusa preamp (Tucker-Davis Technologies Inc.) and from the preamp to an RZ5 Bioamp Processor for signal processing (Tucker-Davis Technologies Inc.). Once the rat was awake and moving, recordings were taken for 10 min at a sampling rate of 25 kHz using the commercially available Synapse Software v98 (Tucker-Davis Technologies Inc.) with a built-in 300–3000 Hz bandpass filter. Using Plexon Offline Sorter (Plexon Inc., Dallas, TX, USA), data were analyzed to detect single unit activity and assess MEA performance. Data were digitally referenced to the common average across electrodes to remove artifacts because of animal movement, biological processes, or any other common mode signal. Individual spikes were detected using a −4 σ standard deviation cutoff from the mean. Any existing artifacts were removed by setting cutoffs for amplitude (+/− 300 µV) and by removing spikes that occurred on more than five consecutive electrodes. Signals concurrently presented on over five electrodes can usually be identified as motion artifacts from the animal moving or grooming during recording. Motion and other artifacts identified were removed. Automated K-means scanning was conducted to sort and cluster spikes into individual single units and manually verify [38].

### 2.5. Neurophysiological Analysis

Additional calculations were conducted using MATLAB R2021a (Mathworks, Natick, MA, USA). Peak-to-peak voltage (Vpp) was defined as the voltage range from peak to trough of each waveform. The noise was calculated as the root mean square of the electrodes after removing spikes. The signal-to-noise ratio (SNR) was calculated by dividing the Vpp of the ensemble unit waveform by the noise for each unit. The spike rate was defined as the inverse of the median interspike interval per unit as recorded in the Plexon Offline Sorter. Recording data from individual MEA devices that registered multiple active electrodes were averaged together to determine average values for Vpp, noise, SNR, and spike rate for single recording sessions. Recording data were binned into three distinct intervals as previously defined by the normal progression of the foreign body response [58]: acute neuroinflammation up to 5 weeks post-implantation, the sub-chronic response between weeks 6 and 11, and finally, the chronic neuroinflammatory response during weeks 12 and onward. For grouping Vpp, noise, SNR, and spike rate, the values of each individual electrode were averaged together within the time phase and group (DMF vs. control). For example, all Vpp values for electrode 1 of the DMF animals in the acute phase were averaged together to obtain a singular value. This was repeated for each electrode and each time phase between groups. To calculate the number of units per active electrode, the sum of units for each electrode of a device in that time phase was recorded and divided by the number of weeks in that specific time phase. This results in an average number of units per active electrode per device for all weeks in a specific time phase in each group. Since an electrode may be active for one week and inactive for others within the same time phase, there are often electrodes with less than one active unit per electrode. For all recording metrics, the sample size is 125 electrodes for acute DMF and 121 electrodes for acute control, 141 electrodes for sub-chronic DMF and 137 electrodes for sub-chronic control, and 124 electrodes for chronic DMF and 144 electrodes for chronic control. To determine the proportion of active electrodes in each group, the total number of active electrodes (electrodes recording at least one unit) was summed and divided by the total possible electrodes (16 electrodes per device × 11 animals per group × 1 week) on a week-by-week basis. The active and total electrodes were summed across weeks, making up each time phase when grouped into acute, sub-chronic, and chronic phases. Doing so leaves a total sample size of 880 for acute (11 animals × 16 electrodes × 5 weeks), 1056 for sub-chronic (11 animals × 16 electrodes × 6 weeks), and 880 (11 animals × 16 electrodes × 5 weeks) for chronic. When considering individual-week proportional comparisons, the sample size is determined by the total number of electrodes multiplied by the number of animals (*n* = 176 per week for both DMF and control).

### 2.6. Histological Tissue Processing 

After reaching the 16-week endpoint for the study, each animal received an IP anesthetic injection of ketamine (160 mg/kg) and xylazine (20 mg/kg). Sufficient anesthetic depth was determined via toe pinch and achieved when there was no associated animal reaction following the pinch. After full anesthetization, a vertical incision was made just below the xiphoid process up the abdomen to expose the abdominal cavity. A second lateral incision was made across the abdominal cavity, followed by a vertical incision upward on each side of the rib cage. The rib cage was held open to expose the pleural cavity and the heart, and the diaphragm was cut with scissors. Afterward, the sternum was clamped with a hemostat to keep the heart and major blood vessels exposed, and a small incision was made in the left ventricle of the heart. A gavage was inserted into the aorta through the left ventricle and clamped to stay in place. To allow blood and perfused liquid to flow out, the right atrium was snipped, and the rat was perfused with 400–500 mL of 1× PBS (1× PBS, Invitrogen, Carlsbad, CA, USA) at 75–100 mL/min. This was carried out until the liver was flushed beige and the perfused liquid flowing out of the heart was clear. Directly after, 400–500 mL of 10% buffered formalin (Fisher Chemical, Waltham, MA, USA) was perfused throughout the animal, and once complete, the brain was extracted following decapitation. After extraction, the brain was placed in refrigerated 10% buffered formalin for 24 h, followed by a 10% sucrose solution (10 g sucrose dissolved in 100 mL 1× PBS, Sucrose from Millipore Sigma, Burlington, MA, USA) in 1× PBS for 24 h, 20% sucrose (20 g sucrose dissolved in 100 mL 1× PBS) for another 24 h, 30% sucrose (30 g sucrose dissolved in 100 mL 1× PBS) for 48 h, and then a final fresh solution of 30% sucrose (30 g sucrose dissolved in 100 mL 1× PBS) until the brains were frozen in the Optimal Cutting Temperature compound (OCT, Sakura Finetek USA Inc., Torrance, CA, USA, #25608-930) for sectioning. Following freezing, to prepare for immunohistochemistry, each brain was sliced at 20 μm section thickness on a cryostat before being placed on microscope slides (SuperFrost Plus, FisherBrand, Hampton, NH, USA). 

### 2.7. Immunohistochemical Staining

Immunohistochemical protocols were followed utilizing the Bond RX automated staining system (Leica Biosystems, Wetzlar, Germany) and our current standard laboratory procedures [18,60,61,62]. The tissue was stained to analyze the levels of astrocyte activity, microglia/macrophage activity, neuron levels, and BBB permeability. Brain tissue was thawed in a humidity chamber at room temperature for one hour, followed by a brief rehydration in 1× PBS to remove any residual OCT compound. The tissue was then loaded into the Bond RX system for automated staining. A proprietary Bond RX detergent wash buffer was applied to tissue to permeabilize cells and tissue for staining cells and molecules. A 10-min heat-induced epitope retrieval step (HIER) was carried out on each slide using a proprietary sodium citrate-based solution at 80 °C. After detergent washing and HIER, one-half of the tissue was incubated for 30 min with rabbit anti-immunoglobulin G (IgG, 1:100, Bio-Rad, Hercules, CA, USA, Catalog #618501) to visualize BBB permeability. Immediately after, the same set of tissue was then rewashed with detergent before incubation with mouse anti-neuronal nuclei (NeuN 1:250, Millipore Sigma, Burlington, MA, USA, Catalog #MAB3477) for neuron-level analysis. The other half of the tissue was incubated for 30 min with a combination solution containing rabbit anti-glial fibrillary acidic protein (GFAP, 1:500, Agilent Dako, Santa Clara, CA, USA, Catalog #Z0334429-2) to visualize astrocytes and mouse anti-CD68 (CD68, 1:100, Millipore Sigma, Burlington MA, USA, Catalog #MAB1435) to visualize activated microglia and macrophages. After incubation, both tissue sets were removed from the Bond RX automated stainer and placed back in a humidity chamber. Subsequently, the tissue was incubated at room temperature for two hours in a secondary antibody solution. The buffer was made up of fluorescent AlexaFluor secondary antibodies 488/594 (1:1000, Invitrogen, Waltham, MA, USA, Catalog #PIA32723, and #A11037, respectively) to visualize markers along with 4′6-diamidino-2-phenylindole (DAPI) (1:3600, 10.9 mM, Invitrogen, Waltham, MA, USA, Catalog #D3571) to visualize cell nuclei. Lastly, tissue was washed with dH_2_O, and excess dH_2_O was wiped off before slides were mounted with coverslips using a mounting medium (Fluoromount-G, Southern Biotech, Birmingham, AL, USA, Catalog #010001). For each group, there was a sample size between 4 and 7. The variation in sample size was due to defective microscope slides leading to a loss in brain tissue for staining.

### 2.8. Imaging and Analysis 

Stained tissues on slides were scanned under the 20× objective of the Axioscan.Z1 microscope (Zeiss Inc., Oberkochen, Germany) and saved as .CZI files. The exposure time was optimized for individual fluorescent markers and was kept consistent throughout the study. In each .CZI file, regions around the implantation hole (9500 × 9500 pixels) were cropped and converted into 16-bit .TIFF (Tag Image File Format) images for intensity analysis. A set of .PNG (Portable Network Graphics) images containing NeuN and DAPI channels were also exported from the .CZI files for neuron counting. For analysis of stain intensity, a custom MATLAB (Mathworks, Natick, MA, USA) program, SECOND, was used to outline the implant hole and mask artifacts from the subsequent analysis [63]. Using a custom Python script, the intensities of fluorescent markers for glial fibrillary acidic protein (GFAP), CD68 (Cluster of Differentiation 68), and Immunoglobulin G (IgG) in the unmasked parts of .TIFF images were quantified and binned into 50 µm intervals based on distance from the implantation site. All pixels within 700 µm from the implantation site were included in the analysis. Intensities in all bins were normalized to the average intensity in the 650–700 µm bin, which was defined as the background. Due to the regular activity of astrocytes in the brain, the normalization constant for GFAP was adjusted to 1. Given that we do not expect IgG and CD68 staining in healthy tissue, the background intensity level for these markers was adjusted to 0. To count neurons stained by NeuN, the Cellpose algorithm segmented the neurons with DAPI (4′,6-diamidino-2-phenylindole) as the reference nuclei channel [64]. The cyto2 model for neuron counting was pre-trained on an independent set of images with default parameters. Another custom Python program was used to calculate the neuron density from the raw outputs of Cellpose. Using the same program, all density data were binned into 50 µm intervals, up to 500 µm away from the implantation hole. For each sample, the density of each bin was normalized to the value of 400–450 µm bin, which was defined as the baseline neuron density. All histological data were shown in the 300–350 µm range due to a lack of meaningful differences beyond this distance. No outliers were removed during the analysis. The only removal or exclusion conducted was on tissue that had clear tears, holes, or blurry images to prevent artifacts. 

### 2.9. Bulk Gene Analysis

A second cohort of 13 animals (7 control and 6 DMF) were implanted with a non-functional “dummy” silicon shank matching the size and shape of the functional implant to evaluate the neuroinflammatory response at a sub-chronic timepoint (7 weeks) within the range of increased recording performance. Surgical differences are outlined in Section 2.3. After seven weeks, the rats were perfused with 1× PBS, followed by 30% sucrose in 1× PBS, and the brains were frozen in OCT after extraction. Each frozen brain was sectioned in a cryostat at 150 µm thickness to isolate brain tissue around the implant site. Next, a 500 µm radius biopsy punch was centered over the implant site to isolate tissue radial to the implant. Biopsy punches were taken throughout the depth of the implant (~8 punches for a total of 1200 µm depth). Brain tissue from the biopsy punch was placed in sterile, nuclease-free tubes containing homogenization beads (#SKU 19-627, Omni International, Kennesaw, GA, USA). Following tissue isolation, samples were homogenized, and RNA was extracted using the Case Western Reserve University Translational Shared Resource Core Facility. Once RNA was extracted, samples were run on the NanoString nCounter for bulk gene analysis using a customized panel of 152 genes (Table 1). The custom panel of genes was selected based on available oxidative stress markers, previously analyzed genes upregulated after MEA implantation, and previously identified housekeeping genes for rat brain tissue (Table 1) [27,65,66,67,68]. Isolated RNA was hybridized with NanoString capture and reporter probe sets, which bind to the 152 RNA sequences of interest using complementary sequences. These hybridized samples were then suspended in a clear cartridge. The capture probes secure the RNA in a stable position to align the reporter probes. The reporter probes consist of a fluorescent sequence unique to each gene of interest, which is then scanned by the nCounter. The NanoString nCounter then outputs raw counts of all 152 RNA sequences scanned within each sample.

### 2.10. Statistical Analysis

Excel (Microsoft Corporation, Redmon, WA, US), R Studio 2022.7.1+554 (RStudio, PBC, Boston, MA, USA), and GraphPad Prism (Dotmatics, Boston, MA, USA) were used to conduct data analysis, graphing, and statistical measurements. Outliers were identified based on 3 median absolute deviations away from the median and removed using the UnivOut1 package in R Studio. A one-tailed proportions z-test was used for calculating statistical differences in the proportion of active electrodes between groups for the acute, sub-chronic, and chronic phases. Additional recording metrics were compared within and across acute, sub-chronic, and chronic neuroinflammatory phases using a Kruskal-Wallis test followed by a Benjamini–Krieger–Yekutieli test for multiple comparisons for non-normal distributions to increase statistical power and reduce type I errors. Statistical comparisons for DMF vs. control were only conducted within the same time point (acute DMF vs. acute control, sub-chronic DMF vs. sub-chronic control, chronic DMF vs. chronic control). No comparisons were made for acute control vs. chronic DMF, acute control vs. sub-chronic DMF, etc. due to a lack of importance. Comparing the performance of the treatment to the control at a different time point does not provide relevant information on efficacy. A standard Student’s *t*-test was performed for each distance interval for immunohistochemical analysis. In all cases, statistical significance was defined at *p* < 0.05. Bar plots using error bars with the standard error of the mean (SEM). For recording data box plots, whiskers represent minimum and maximum values, the box represents the first and third quartiles of the data, and the horizontal line indicates the median. All recorded numerical data are represented in the text as the mean ± SD. Bulk RNA expression data were analyzed using the NanoString nSolver software as previously described by our lab [65,67,68]. The raw expression counts were normalized with positive and negative control probe counts to account for assay efficiency and housekeeping genes that normalize to the amount of RNA collected per sample. Following normalization, a differential expression analysis was performed to determine how gene expression changes with DMF treatment. Any gene with less than 20 counts in 85% of the samples was removed. The expression ratio was plotted on a log_2_ scale and called the Log_2_FoldChange. Expression of Log_2_FoldChange > 1 indicates a two-fold increase, whereas Log_2_FoldChange < −1 indicates a two-fold decrease. A 2-tailed, unequal variance *t*-test was performed for each gene. A Benjamini–Hochberg correction using a false discovery rate of 0.05 filtered out random significance due to the many genes tested.

## 3. Results

### 3.1. Effect of DMF on MEA Performance and Single-Unit Recordings

**Figure 2:**
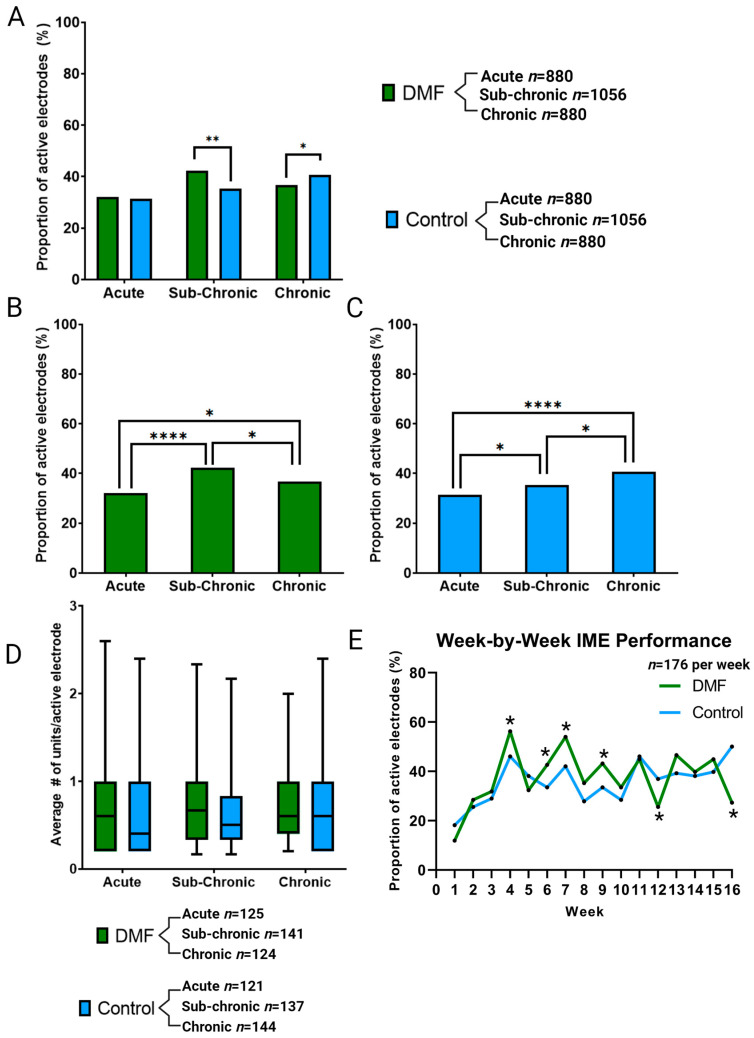
Neurophysiological recording activity to evaluate the (**A**) proportion of active electrodes (AEY %) on each recording device for comparison between DMF-treated animals and control animals. Additional direct comparisons from the same data set across neuroinflammatory phases for DMF-treated animals (**B**) or control animals (**C**). (**D**) Evaluation of the number of units detected per active electrode compared between DMF and control. (**E**) Week-by-week comparison of MEA recording performance for each group. Significance is denoted as *p* < 0.05 = *, *p* < 0.01 = **, *p* < 0.0001 = ****. No symbol indicates a lack of statistical significance. No comparisons were made between DMF and control at differing time points. The sample size for (**A**–**C**) is determined by the total number of electrodes multiplied by the number of weeks in each phase and the number of animals in each group. The sample size for (**D**) is determined by the number of active electrodes multiplied by the number of weeks in each time point and the number of animals in each group. Sample size for (**E**) is determined by the total number of electrodes multiplied by the number of animals in each group on a week-by-week basis.

Figure 2A illustrates the proportion of active electrodes in acute (Weeks 1–5), sub-chronic (Weeks 6–11), and chronic (Weeks 12–16) phases. At the acute phase, DMF (32% active electrode yield) showed no significant difference compared to controls (31% active electrode yield). Within groups, DMF-treated animals (*n* = 11) demonstrated a significant but temporary increase in the active electrode yield with higher recording performance at the sub-chronic phase, only to decline in the chronic phase (Figure 2B). Control animals (*n* = 11) also improved from acute to sub-chronic to chronic (Figure 2C). However, the active electrode yield recovered in the chronic phase for control animals (Figure 2C). The steady increase in the proportion of active electrodes in the control animals may result from stabilization after the wound healing response in the acute phase and BBB stabilization during the late sub-chronic phase. The differential trends within treatment modalities led to differences in the comparison between treatment groups at both the sub-chronic and chronic phases (Figure 2A). Specifically, during the sub-chronic phase, DMF treatment (42% active electrodes) shows a significant improvement in the proportion of active electrodes (*p* = 0.0005) compared to control animals (35% active electrodes). The sub-chronic improvement in active electrode yield for DMF-treated animals was lost during the chronic phase, as DMF-treated animals declined in performance (37% active electrodes) while control animals continued to improve to a statistically higher proportional yield (*p* = 0.043; 41% active electrodes; Figure 2A). 

Units per electrode were compared within and between groups across neuroinflammatory phases for both DMF-treated and control animals. Despite changes in the active electrode yield between and within treatment groups, the average number of units per electrode site remained indistinguishable across all inflammatory phases (Figure 2D, no significant differences, *p* > 0.05). The week-by-week comparison (Figure 2E) shows the improvement of DMF compared to control for the acute phase at only week 4 and improvements in the sub-chronic phase in weeks 6, 7, and 9. We observe significant declines at weeks 12 and 16 during the chronic phase. Week 7 specifically showed the largest improvement between DMF and control at 11.9%. 

**Figure 3:**
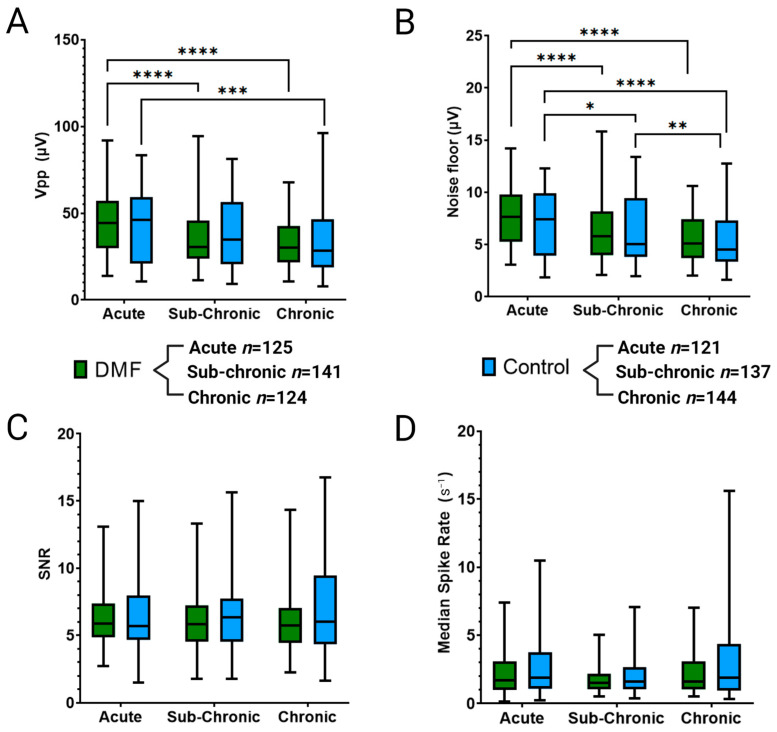
Additional neurophysiological metrics to evaluate recording performance include (**A**) peak-to-peak voltage of signals (Vpp), (**B**) noise levels for each electrode, (**C**) signal-to-noise ratio, and (**D**) the median spike rate for activity. Significance is denoted as *p* < 0.05 = *, *p* < 0.01 = **, *p* < 0.001 = ***, and *p* < 0.0001 = ****. No symbol indicates a lack of significance. No comparisons were made between DMF and control at differing time points. Sample size is determined by the number of active electrodes multiplied by the number of weeks in each time point and the number of animals in each group.

As shown in Figure 3A, Vpp was comparable between DMF-treated animals and control groups across acute (44.8 ± 17.2 µV for DMF and 42.5 ± 20.2 µV for control), sub-chronic (34.6 ± 14.9 µV for DMF and 38.0 ± 19.8 µV for control), and chronic (32.7 ± 12.9 µV for DMF and 33.9 ± 19.1 µV for control) phases. The DMF treatment resulted in a significant decline in Vpp from acute to sub-chronic (*p* < 0.0001) and acute to chronic (*p* < 0.0001) phases (Figure 3A). Control animals showed a similar decline from acute to chronic (*p* = 0.0003, Figure 3A). 

A comparison of noise levels showed no significant differences between treatment groups for acute (7.5 ± 2.6 µV for DMF and 7.0 ± 3.0 µV for control), sub-chronic (6.1 ± 2.5 µV for DMF and 6.3 ± 3.1 µV for control), and chronic (5.7 ± 2.3 µV for DMF and 5.4 ± 2.7 µV for control) neuroinflammatory phases (Figure 3B). DMF-treated animals showed a significant decline in noise from acute to sub-chronic (*p* < 0.0001) and from acute to chronic (*p* < 0.0001) phases (Figure 3B). Control animals also showed a significant decline in noise from acute to sub-chronic (*p* = 0.0343), acute to chronic (*p* < 0.0001), and sub-chronic to chronic (*p* = 0.0097, Figure 3B). Due to a lack of differences in Vpp and noise levels, the SNR (Figure 3C) for both treatment groups was not statistically different (acute: 6.2 ± 2.0 for DMF and 6.5 ± 2.9 for control, sub-chronic: 6.2 ± 2.4 for DMF and 6.6 ± 2.9 for control, chronic: 6.3 ± 2.6 for DMF and 7.0 ± 3.7 for control). There were no significant changes in SNR between phases, most likely due to similar declines in Vpp and noise levels from acute to sub-chronic to chronic phases (Figure 3C). Spike rate (Figure 3D) showed no significant changes between control and DMF-treated within a given phase: acute (2.2 ± 1.5 for DMF and 2.7 ± 2.1 for control), sub-chronic (1.7 ± 0.9 for DMF and 2.0 ± 1.3 for control), and chronic (2.2 ± 1.5 for DMF and 3.4 ± 3.6 for control). Additionally, no significant changes were observed across phases for a given treatment group.

### 3.2. Effect of DMF on Tissue Health and Neuroinflammation

#### 3.2.1. Chronic Inflammation and Blood-Brain Barrier Permeability

**Figure 4:**
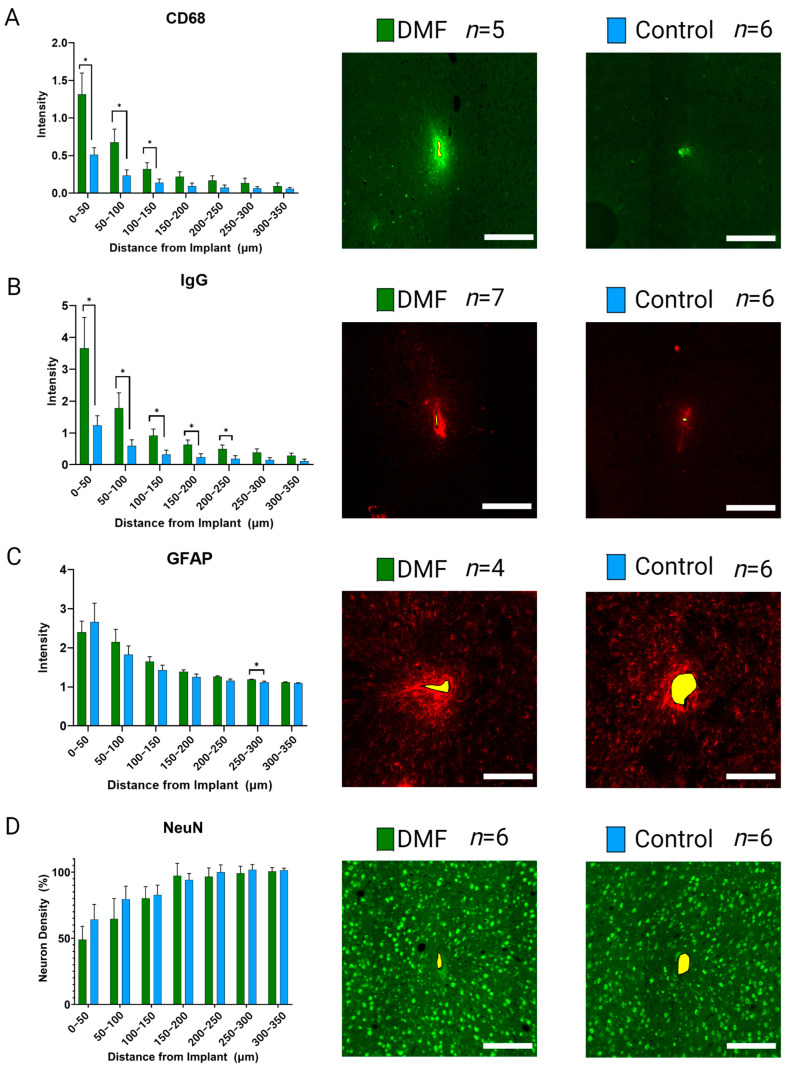
Immunohistochemical evaluation of the implant site 16 weeks post-implantation. Representative images for each stain are included to the right of the quantification plots. (**A**) CD68 expression indicating the presence of active microglia and macrophages; (**B**) IgG expression indicating BBB stability; (**C**) GFAP activity indicating the presence of hypertrophic astrocytes and glial scar formation; and (**D**) Neuronal Nuclei (NeuN) expression to evaluate the density of neurons around the implant. Scale bars = 200 µm. Significance is denoted as *p* < 0.05 = *. No symbol indicates a lack of significance. Variation in sample size is a result of a set of defective microscope slides leading to a loss in brain tissue when staining.

Immediately after implantation, microglia and macrophages migrate to the implant site, activate to clear debris, and combat the foreign body presence of the MEA [6]. Chronic activation results in the overproduction of ROS and perpetual neuroinflammation, contributing to the failure of the MEA [33,69,70]. Figure 4 shows the neuroinflammatory response to implanted MEAs in DMF-treated and control animals following 16 weeks of implantation. DMF-treated animals have significantly (*p* < 0.05) higher expressions of active microglia/macrophage markers (CD68; Figure 4A) within 150 µm of the implant compared to controls. Increased CD68 expression is indicative of an elevated neuroinflammatory response [14]. Furthermore, DMF-treated animals demonstrated significantly higher immunoglobulin (IgG; Figure 4B) levels within 250 µm of the implant site, indicating higher BBB permeability. Such results suggest chronic DMF usage may weaken the BBB, allowing more macrophages to infiltrate the implant site and further perpetuate the neuroinflammatory response. Staining for glial-fibrillary acidic protein (GFAP; Figure 4C) showed no statistical difference within the immediate recording area (within 150 µm) from the implant site, indicating no difference in glial scarring at 16 weeks post-implantation. The significance observed at 250–300 µm suggests that the DMF group has higher astrocytic activity in that region. However, the difference in intensity (1.19 for DMF vs. 1.12 for control) is minimal, suggesting that significance is simply due to the low variance within animals. At 250–300 µm, we do not usually expect a biological difference due to the implant unless the closer regions also display significant differences in expression.

#### 3.2.2. Neural Health and Viability

Following MEA implantation, neuronal populations demonstrate a trend of reduced density, typically measured with neuronal nuclei (NeuN) staining, in the 200–250 µm closest to the implant surface [6]. Due to the antioxidant and neuroprotective effects of DMF previously demonstrated in the literature [41,42], neuronal density was hypothesized to improve over 16 weeks compared to control animals, leading to improvements in MEA recordings. However, no significant change was seen in normalized neuronal nuclei (NeuN; Figure 4D), indicating a relatively stable population of neurons compared to controls. Such results show that regular DMF treatments did not increase the ability to prevent neuron loss 16 weeks post-implantation of MEAs.

### 3.3. Bulk Gene Expression Analysis

**Figure 5:**
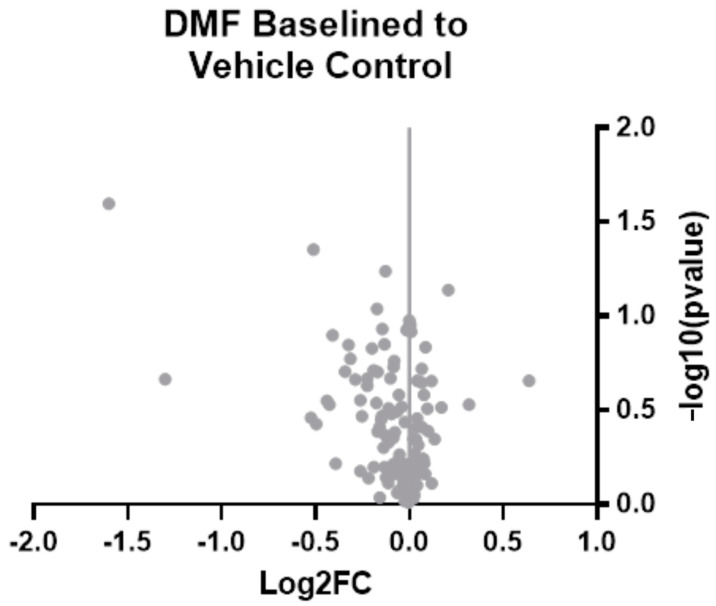
Bulk gene analysis 7 weeks post-implantation on 152 oxidative stress and neuroinflammatory genes associated with neural implants. Log_2_ (fold change) is based on DMF values compared to the control using differential expression analysis with a 2-tailed unequal variance *t*-test for each gene. Grey points indicate no significant difference compared to the control. *n* = 6 for DMF-treated; *n* = 7 for control.

To further understand the neuroinflammatory and oxidative stress responses in the brain, we performed experiments with an additional cohort of animals. In this additional cohort, animals were given treatment for seven weeks. Week 7 was chosen due to the most significant margin of improvement observed in MEA recording performance in the functional implant cohort (Figure 2E). In this second cohort, six rats were treated with DMF, and seven were treated with the control vehicle. Rather than using 4–6 histological markers and performing immunohistochemistry again, we developed a custom gene expression panel with 152 genes associated with neuroinflammatory and oxidative stress pathways (Table 1). Here, our results from bulk gene analysis based on log_2_ (fold change) of DMF-treated animals compared to control animals suggest no significant differences between groups after correction (Figure 5). 

## 4. Discussion

In previous studies, we observed an acute average electrode yield in control animals that was higher than that displayed for our controls here. It is important to note that the control animals here were administered a daily diluent gavage. While not treated with antioxidants, they were treated and handled more regularly than our historical control animals, which could account for a lower active electrode yield than traditional control animals [38]. At sub-chronic time points (weeks 5–11), DMF-treated rats displayed significantly improved recording performance compared to vehicle control (Figure 2 and Figure 3). Specifically, week 7 showed the most significant margin of improvement in DMF compared to controls (Figure 2E). However, DMF-treated animals began to decline at chronic time points (12+ weeks) and were significantly worse than control. To understand why such a reversal of expected performance occurred, we measured the histological outcomes of the brain at 16 weeks (Figure 4) and the genetic analysis of 152 neuroinflammatory and oxidative stress-related genes at seven weeks (Figure 5). 

The immunohistochemical evaluation of the brain tissue adjacent to the MEA at 16 weeks post-implantation showed a significant increase in IgG and CD68 levels immediately around the implant (0–200 µm) for DMF-treated animals compared to control animals. IgG is naturally found in high abundance, circulating in the bloodstream but not in the brain [71]. CD68 expression is a marker for increased microglia and macrophage activation to an inflammatory phenotype, indicating an elevated neuroinflammatory response [14]. Therefore, the abundance of IgG found at the MEA-tissue interface at 16 weeks indicates less stability in BBB integrity at some point post-implantation. There is debate in the literature about whether IgG expression is a static or dynamic measure of BBB permeability and whether IgG is cleared or accumulates from/at the implant site. Additionally, higher levels of CD68 expression surrounding the implants in the DMF-treated group indicate increased microglia and macrophage activation (Figure 4A,B). 

BBB integrity and CD68 expression may follow similar trends due to the prevalence of infiltrating macrophages coming from the bloodstream into the implant site [72,73,74]. Several labs have indicated a self-perpetuating relationship between activated microglia and macrophages and BBB integrity, partly due to the secretion of reactive oxygen species by activated microglia and macrophages, which compromises BBB integrity [6,11,26,75]. We have previously shown that blood-derived macrophages are responsible for a large portion of mouse models’ neuroinflammatory responses to MEAs [17,18]. Additionally, multiple studies have linked IgG expression to MEA recording performance [7,76,77]. 

Therefore, poor BBB stability and resulting macrophage infiltration may explain the reversal of sub-chronic improvements in recording performance and the subsequent lack of chronic improvements in neurophysiological recordings for DMF-treated animals. Elevated neuroinflammation during the chronic phase could mask the initial improvements in recording performance for DMF-treated animals. 

To understand why sub-chronic recording improvements occurred, we set up additional animals to be sacrificed at the 7-week time point, where the greatest weekly difference in active electrode yield was seen (Figure 2E). Bulk gene analysis of oxidative and neuroinflammatory genes at seven weeks post-implant did not reveal any significant changes between DMF and control groups (Figure 5). It was unexpected to see a lack of differences given DMF’s antioxidant capabilities and the recording improvements observed at that time. However, changes to gene expression may be a predictive indicator for functional experiments like microelectrode recording differences. A future study could look at gene expression in preceding weeks (for example, Weeks 5 and 6 here) to observe if molecular-level changes in the neuroinflammatory process take time to impact recording observations and MEA performance. Additionally, further analysis of gene panels may be warranted, as those involved in cellular structure and synaptic transmission, to name a few, have been linked to neural inflammation [78]. With new spatial transcriptomic and whole transcriptome analyses becoming more feasible, using a wide range of genes will offer the most insight into mechanistic alterations due to MEA implantation. 

It is essential to discuss that DMF treatments designed to assess the impact on neurological disorders in rodent models have shown neuroprotective and antioxidant effects throughout the study. However, it is worth noting that the referenced studies were designed to end before our chronic phase time point of 16 weeks, with most studies lasting either less than a few weeks (acute) [41,42,45] or not beyond ten weeks (sub-chronic) [47,56]. While we did not uncover the apparent side effects of DMF treatment, as we have noted with daily IP injections of Resveratrol [24], there may be undetected side effects masking the beneficial effects during the chronic phase. For example, regular oral gavage treatments have been linked to adverse health effects due to changes in the gut microbiome and plasma metabolome [79]. While there is growing evidence suggesting that altering gut microbiome composition can alter brain and neuron health through the gut-brain axis [80,81,82,83,84,85,86], in the context of this study, we did not evaluate potential changes in gut microbiome for either control or DMF-treated animals compared to naïve sham animals. To that end, it is essential to point out that both control and DMF-treated animals demonstrated a lower active electrode yield than we have seen in other studies in our labs [38,57,58]. Extensive rat handling and gavage training using a claw-grip technique were conducted prior to the start of the study to minimize the impact on the animal. However, it is possible that decreased recording performance in both groups was a result of unnoticed complications such as elevated stress levels due to regular oral gavage [87,88]. 

Regarding the known mechanism of action for the antioxidative effects of DFM treatment, DMF has been shown to exert neuroprotective and antioxidant effects through its ability to activate the Nrf2 pathway [47,52,56]. Nrf2 is a transcription factor that, when activated, provides anti-inflammatory, cytoprotective, and antioxidant effects to the brain [49,89]. In the gene panel shown in Table 1, Gsta1, Gsta2, KEAP1, and Nqo1 are involved directly in the NRF2 pathway but were not significantly changed when animals were treated with DMF [90]. Although DMF has been shown to act through the Nrf2 pathway, the complete mechanism of DMF activity remains to be determined. DMF has been shown to impact many aspects of the innate and adaptive immune systems, including reductions in absolute lymphocyte counts [52,91,92]. Declines in lymphocyte count are one reason why chronic use of DMF has been linked to an elevated risk of infection [93,94]. No signs of chronic infections were seen in this study. However, with such a broad range of effects and a need for a more precise understanding, DMF may trigger a response in which the BBB needs to heal appropriately over time due to persistent MEA presence, leading to poor chronic results. To that end, we have shown that over-attenuating the innate immune response to MEAs can negatively impact recording performance compared to partial inhibition of the same target [18,21]. Therefore, a smaller or less frequent dose of DMF could facilitate a longer-lasting improvement in MEA performance. Future studies may explore alternative means and dosing regimens for improvements in chronic MEA performance. 

Although the current delivery and dose for DMF suggest a lack of recording improvement in the chronic period of implantation, there may still be utility in using DMF for temporary improvements in MEA performance on the sub-chronic timescale (<12 weeks). Clinically, MEA devices are designed to be “lifetime” solutions for brain-computer interfacing systems. However, there are still many short-term uses for MEAs [95]. Outside of clinical use, sub-chronic improvements in recording performance can also benefit basic science and hypothesis-driven neuroscience studies. Poor recording performance is continuously an issue in signal processing for decoding brain activity into peripheral motor action [6,96,97]. Improving recordings in the sub-chronic phase can aid in the signal decoding process. Additionally, DMF may be one part of a treatment cocktail that utilizes a multipronged approach to mitigate several aspects of the neuroinflammatory response robustly and temporally to implanted MEAs.

## 5. Conclusions

DMF is a widely used therapy for treating relapsing multiple sclerosis due to its neuroprotective and antioxidant therapeutic effects. We show that DMF treatment produces a statistically significant improvement in active electrode yield within a sub-chronic neuroinflammatory phase (5–11 weeks). Though the current formulation and dosing did not provide an indefinite long-term improvement beyond ten weeks post-implantation to declining MEA performance, temporary improvements to neurophysiological recordings are still crucial for short-term brain-machine interface uses and basic science studies. Further optimization of the dosing of DMF or the inclusion of DMF in combination therapies could result in a more robust and comprehensive approach to mitigating MEA failure.

## Figures and Tables

**Table 1:** A complete list of neuroinflammatory and oxidative stress genes of interest that were utilized in this study. Here we list the 152 genes examined in rat brain tissue in this study using a combination of custom genes, preset genes from NanoString, and housekeeping genes in their respective columns. Genes highlighted in yellow are directly involved in the antioxidant NRF2 pathway, with which DMF is hypothesized to interact.

Custom Gene Panel			NanoString Gene Panel					Housekeeping Gene Panel
AIM2	Ercc6	Nr2f6	Abl1	Cycs	Hspb1	Nos1	Tnf	Hprt
ARC	FCER1G	Osgin1	Ager	Ddit3	Htra2	Nos3	Tor1a	Rpl13a
Bdnf	FCGR2B	OSMR	Aif1	Dnm2	Idh1	Nr4a2	Tpm1	Rps18
BLNK	GFAP	Prnp	Akt1	Ep300	Il1r1	Oxr1	Trp53	Sdha
C3	GSTA1	PSMB8	Apoe	Fas	Il6	Park7	Trpm2	Tbp
C3AR1	Gsta2	Ptgs2	App	Fn1	Ins2	Parp1	Txnl1	Ubc
C4A	GSTM2	PTPN6	Atf4	Fos	Ipcef1	Pdgfrb	Ubqln1	
C5AR1	Hmox1	PTX3	Atp13a2	Fxn	Jun	Pink1	Xbp1	
CASP8	Il1b	SCD1	Atp7a	Gnao1	Lpo	Pla2g4a		
CCL1	IL2RG	SERPINA3N	Atrn	Gpr37	Lrrk2	Ppargc1a		
CD14	IRAK4	Sod3	Bad	Gsk3b	Mapt	Psen1		
CD36	IRF7	SPP1	Bcl2	Gsr	Mgmt	Rela		
CD45	ITGAM	Srxn1	Bnip3	Gss	Mmp14	Sirt1		
CD68	KEAP1	TNFRSF1A	Casp3	Gstp1	Mutyh	Sirt2		
CD74	LILRB4A	TNFRSF25	Ccl5	Gucy1b3	Ncf1	Slc8a1		
CD84	MMP12	Txnrd1	Ccs	H2-T23	Nefh	Snca		
CLEC7A	MPEG1	TYROBP	Cdk2	Hdac2	Ngfg	Sod1		
CTSS	Nfe2l2	Vegfa	CIM	Hdac6	Ngfr	Sod2		
DOCK2	Noxa1		Cln8	Hgf	Nme5	Src		
Ehd2	Nqo1		Cybb	Hif1a	Nol3	Stx2		

## Data Availability

The data presented in this study are available upon request from the corresponding authors.
